# The Therapeutic Potential of Mangosteen Pericarp as an Adjunctive Therapy for Bipolar Disorder and Schizophrenia

**DOI:** 10.3389/fpsyt.2019.00115

**Published:** 2019-03-13

**Authors:** Melanie M. Ashton, Olivia M. Dean, Adam J. Walker, Chiara C. Bortolasci, Chee H. Ng, Malcolm Hopwood, Brian H. Harvey, Marisa Möller, John J. McGrath, Wolfgang Marx, Alyna Turner, Seetal Dodd, James G. Scott, Jon-Paul Khoo, Ken Walder, Jerome Sarris, Michael Berk

**Affiliations:** ^1^IMPACT Strategic Research Centre, School of Medicine, Barwon Health, Deakin University, Geelong, VIC, Australia; ^2^Florey Institute for Neuroscience and Mental Health, University of Melbourne, Parkville, VIC, Australia; ^3^Professorial Unit, The Melbourne Clinic, Department of Psychiatry, University of Melbourne, Richmond, VIC, Australia; ^4^Department of Psychiatry, Royal Melbourne Hospital, University of Melbourne, Parkville, VIC, Australia; ^5^Centre for Molecular and Medical Research, School of Medicine, Deakin University, Geelong, VIC, Australia; ^6^Professorial Psychiatry Unit, Albert Road Clinic, University of Melbourne, Melbourne, VIC, Australia; ^7^Centre of Excellence for Pharmaceutical Sciences, School of Pharmacy (Pharmacology), North West University, Potchefstroom, South Africa; ^8^Queensland Centre for Mental Health Research, The Park Centre for Mental Health, Wacol, QLD, Australia; ^9^Queensland Brain Institute, University of Queensland, St. Lucia, QLD, Australia; ^10^National Centre for Register-Based Research, Aarhus University, Aarhus, Denmark; ^11^Centre of Youth Mental Health, University of Melbourne, Parkville, VIC, Australia; ^12^Faculty of Medicine, The University of Queensland, Herston, QLD, Australia; ^13^Metro North Mental Health, Royal Brisbane and Women's Hospital, Brisbane, QLD, Australia; ^14^NICM Health Research Institute, Western Sydney University, Westmead, NSW, Australia; ^15^Orygen Youth Health Research Centre, Parkville, VIC, Australia

**Keywords:** mangosteen pericarp, bipolar disorder, schizophrenia, psychiatry, oxidative stress, inflammation, mitochondria

## Abstract

New treatments are urgently needed for serious mental illnesses including bipolar disorder and schizophrenia. This review proposes that *Garcinia mangostana Linn*. (mangosteen) pericarp is a possible adjunctive therapeutic agent for these disorders. Research to date demonstrates that neurobiological properties of the mangosteen pericarp are well aligned with the current understanding of the pathophysiology of bipolar disorder and schizophrenia. Mangosteen pericarp has antioxidant, putative neuroprotective, anti-inflammatory, and putative mitochondrial enhancing properties, with animal studies demonstrating favorable pharmacotherapeutic benefits with respect to these disorders. This review summarizes evidence of its properties and supports the case for future studies to assess the utility of mangosteen pericarp as an adjunctive treatment option for mood and psychotic disorders.

## Introduction

Serious mental illness, generally defined as disorders with psychotic or high severity symptoms (such as bipolar disorder and schizophrenia), contribute significantly toward disease burden worldwide (1). Importantly, those living with serious mental illnesses often experience suboptimal responses to conventional treatments (2, 3), and treatment options are limited (2, 4). The developmental pipeline for conventional psychiatric medications, historically driven by large pharmaceutical companies, is dwindling (5, 6); as such, the investigation of novel therapeutics is both warranted, and needed. One promising avenue of research is in the potential use of nutraceutical agents, as adjunctive therapies, that target biological pathways known to be dysregulated in neuropsychiatric disorders (7).

This narrative review explores the neurobiological properties and therapeutic potential of an extract derived from the pericarp of *Garcinia mangostana Linn*. (mangosteen) for serious mental illness. Due to its bioactive components and the parallels with the current understanding of the pathophysiology of both schizophrenia and bipolar disorder, the mangosteen pericarp may be or may contain a useful adjunctive therapeutic agent for these disorders. The salient neurobiological targets that overlap in serious mental illness include; oxidative stress, neuroinflammation, neurogenesis and apoptosis, and mitochondrial dysfunction. The potential therapeutic value of mangosteen pericarp will be explored within the context of these factors.

## Bipolar Disorder and Schizophrenia: Shared Physiology

Major neuropsychiatric disorders appear to share much of their basic neurobiology, suggesting that nutraceutical and other agents may have broad utility. Schizophrenia and bipolar disorder exhibit shared genetic and neurocognitive factors and clinical symptoms (8, 9). Similarly, schizophrenia and bipolar disorder have overlapping biological aberrations demonstrated by the use of some drugs to treat both conditions (e.g., atypical antipsychotics) (10).

### Monoamine Disturbances

Monoamines play a critical role in the pathophysiology of bipolar disorder and schizophrenia (11, 12). Glutamatergic dysregulation has also been implicated in the pathophysiology of bipolar disorder (13–15) and schizophrenia (16). Excess mesolimbic dopaminergic activity is implicated in the pathophysiology of psychosis (17). Serotonergic dysregulation has also been implicated in bipolar disorder and schizophrenia including alterations in 5-HT_2A_, 5-HT_1A_, and 5-HT_1B_ receptors in the prefrontal cortex and hippocampus (18). Some common psychotropic medications target these pathways but are not effective for everyone. Therefore, new therapies should aim to address critical biological targets which are not addressed by this common monoamine theory (19), but instead mediate changes through the biological processes outlined from here in.

### Oxidative Stress

Altered oxidative biology in serious mental illness is indicated by a reduction in antioxidant levels, including glutathione and glutathione transferase (20, 21) and increased reactive oxygen species (ROS) and reactive nitrogen species (RNS) (20). There may also be a rise in oxidative stress markers such as malondialdehyde (MDA) and thiobarbituric acidic reactive substances (TBARS). Redox markers (20), including nitric oxide, superoxide dismutase, catalase, and glutathione peroxidase are altered in serious mental illness (20, 22, 23). It has been suggested that the variability in redox markers may to some extent be due to differences between early and late stages of the disorder (19).

The high levels of oxidative stress may, in part, originate in mitochondria and are associated with mitochondrial dysfunction (19). Aberrations in neurotransmitters such as glutamate are also associated with altered redox state and this ties into changes in monoamines seen in serious mental illness (13). Targeting redox imbalance has been shown to be a useful therapeutic pathway, exemplified by agents such as N-acetyl cysteine that have conferred some benefits in schizophrenia and bipolar disorder (20, 24–27).

### Inflammation and Neurogenesis

In both schizophrenia and bipolar disorder, there is evidence of raised inflammatory cytokines both in the central nervous system and peripheral circulation (21, 28–32). The effects of neuroinflammation include lowering mitochondrial energy generation, increased free radicals and lipid peroxidation and increased neuroexcitation which may lead to neurodegeneration and apoptosis through raising intracellular calcium and glutamate levels (21, 33, 34). Inflammation can also lead to higher levels of NO being produced by inducible nitric oxide synthase (iNOS) (35). A recent meta-analysis found that acute illness in schizophrenia was associated with elevated levels of the peripheral proinflammatory cytokines interleukin (IL) 6 and tumor necrosis factor alpha (TNF-α), and elevated levels of cytokine receptor antagonist (IL-1Ra) and soluble cytokine receptor (36). In chronically ill patients, peripheral IL-6, IL-1β, and soluble cytokine receptor levels were persistently elevated (36).

Serious mental illnesses are also associated with higher rates of programmed cell death or apoptosis than healthy controls (37, 38), with irregularities in apoptotic and metabolic markers observed in schizophrenia (39). For example, evidence indicates activated apoptotic programmed cell death pathways in the anterior cingulate cortex and hippocampus of patients with schizophrenia (40). Alterations in neurotrophins, which protect against neuronal apoptosis, have been reported in bipolar disorder. For example, brain derived neurotrophic factor, B-cell lymphoma 2 (bcl-2), and vascular endothelial growth factor (VEGF) are decreased during acute phases of bipolar disorder (both mania and depression) (21).

Mitogen-activated protein kinases (MAPK) regulate cell survival and apoptosis via gene expression, cell proliferation, cell survival, and death, and thus, are important for neuronal plasticity (37). Other MAPKs, including p38 and c-Jun N-terminal (JNK), are also possible mediators of mitochondrial-induced apoptosis (41). Neuroleptic medications used in bipolar disorder and schizophrenia (e.g., olanzapine and haloperidol) can activate the MAPK pathway (42).

Heightened neuroinflammation, and indeed microglial activation are associated with inhibition of neurogenesis (43, 44). Some evidence suggests that altered neurogenesis, particularly in the hippocampus, occurs in schizophrenia. For example, a significant reduction in Ki67+ cells (a marker for cell proliferation) was observed in post-mortem hippocampal tissue of patients with schizophrenia (45, 46). Altered postnatal neurogenesis in the striatum was also proposed as an explanation for the dopaminergic deficits commonly reported in schizophrenia (43), in addition to gross hypodopaminergia in the frontal cortex, and hyperdopaminergia in the striatum (16).

Adjunctive treatments targeting inflammation (and consequently neuroprotection), for example celecoxib (COX-2 inhibitor) and minocycline (tetracyclic antibiotic), have shown some efficacy in treating schizophrenia and bipolar disorder [see Müller (47); Sommer et al. (48) for discussion]. Other adjuncts with anti-inflammatory effects, including aspirin, *N*-acetylcycteine, and estrogen modulating treatments were also shown to have potential efficacy (48–51).

### Mitochondrial Dysfunction

Mitochondria are essential contributors to cellular energy metabolism, synaptic transmission and neuronal growth and are involved in oxidative stress and apoptotic pathways. Overproduction of ROS can cause mitochondrial dysfunction by damaging mitochondrial DNA and mitochondrial respiratory chain (leading to a reduction in energy production). Lipid peroxidation can also occur due to ROS and increase the mitochondrial membrane permeability leading to a disruption in Ca^2+^ homeostasis (52). Elevation in intracellular Ca^2+^ levels can cause neuronal degeneration and cell death and can lead to the production of superoxide ion radicals, forming a vicious cycle (52, 53). Differences in the size, shape, and distribution of mitochondria has been reported in post-mortem prefrontal cortex of participants with bipolar disorder compared to healthy controls (54). There is a shift toward glycolysis within the mitochondria which is associated with an impairment of oxidative phosphorylation with lactate accumulation and decreased energy production (55).

Accumulating evidence suggests that differences in mitochondrial abundance, function, and morphology are associated with the onset and pathophysiology of schizophrenia (56, 57). Post-mortem studies of schizophrenia patients have reported region-specific differences in mitochondrial abundance, localization, size, and function across a number of cell types and brain regions [see Roberts (58) for review]. There is also some evidence to suggest a link between schizophrenia symptoms and mitochondrial pathology in the periphery, for example altered microstructure as well as a decreased density of mitochondria in blood lymphocytes, corresponding to dysregulated energy metabolism (59, 60). Medications targeting mitochondria, such as L-acetyl-carnitine (61) and methylene blue (62, 63), have demonstrated therapeutic utility as antidepressants, antipsychotics, and mood stabilizers.

In bringing together the aforementioned relevant information regarding the known pathophysiology of serious mental illness and the known bioactivity of mangosteen pericarp, this review sets the scene for exploring the potential use of mangosteen pericarp for the treatment of serious mental illness. The wide variety of pathophysiological targets discussed, likely combined, may highlight one of the most important and still unsolved problems in the development of psychotropic medications. New developments targeting these pathways in combination, may help to improve treatment response and fill the gap left by conventional treatments.

## *Garcinia Mangostana Linn*. (Mangosteen) Pericarp

*Garcinia mangostana Linn*, more commonly known as mangosteen, is a tropical fruit affectionately referred to as the “Queen of the Fruits” (64, 65). The flesh of the fruit is contained within a husk (pericarp). Mangosteen pericarp has historically been used for its antimicrobial effects in South East Asia to treat skin infections, wounds and dysentery (64). The mangosteen pericarp contains at least 50 different bioactive compounds including polyphenol-subclasses, xanthones and catechins (64). Several of these compounds are reported in this review, including α-mangostin, γ-mangostin, gartanin, 8-deoxygartanin, garciniafuran, garcinone C, and garcinone D (66), 7-O-demethyl mangostanin (67), mangostenone F (68, 69), and mangostenone G (69). Compared to the edible aril part of the fruit, the pericarp contains 10 times more phenolic compounds and 20 times more antioxidant activity (70). It is noteworthy that xanthones are tricyclic compounds and their biological activities might be associated with this chemical structure (64). The most prominent xanthones in the mangosteen pericarp are α-mangostin and γ-mangostin (64). There have been many reports of the potential benefits of xanthones of the mangosteen pericarp, including properties that are antioxidant, anti-inflammatory and anti-apoptotic (64, 71). The properties of mangosteen pericarp have been summarized in Table 1.

**Table 1:** Summary of neurobiological activity of *Garcinia mangostana Linn*. (mangosteen).

**Paper**	**Mangosteen compound**	**Pathway/marker**	**Method**	**Interpretation**
Marquez-Valadez et al. (72)	α-mangostin	↓TBARs ↓ mitochondrial dysfunction	Rats received pro-oxidant agents: ferrous sulfate, quinolinic acid and 3-NP (*n* = 60)	Antioxidant properties, reduced mitochondrial dysfunction
Marquez-Valadez et al. (73)	α-mangostin CH2Cl2–MeOH (dichloromethane) solution extraction	↓GSH ↓GPx ~ Glutathione S-transferase	*In vitro* Rats administered ferrous sulfate, or 3-NP, in addition to α-mangostin and compared to control of only α-mangostin	Selective modulation of GSH system, antioxidant properties
Moongkarndi et al. (65)	α-mangostin Comparing ethyl acetate vs. water extract	↓DPPH (↑ROS scavenging; water extract only) ↓ cancer cell production	*In vitro* Breast cancer (SKBR3) cells	Antioxidant properties
Lee et al. (74)	α-mangostin	↓Bcl-2 ↑Bax ↓MAPk and ERK pathways, ↑apoptosis	*In vitro*—tongue carcinoma cells	
Yang et al. (67)	7-O-Demethyl mangostanin	↑apoptosis	*In vitro* Cancer cells	
Shin-Yu et al. (75)	α-mangostin	↓TBARs ↑ GSH, GPx, glutathione reductase, SOD, and catalase	*In vitro*. High fat diet with mangosteen vs. High fat diet without mangosteen and compared to regular diet control.	Antioxidant properties
Oberholzer et al. (76)	Mangosteen pericarp extract	↓ hippocampal lipid peroxidation	*In vivo*: Flinders sensitive line rats, compared with imipramine (tricyclic antidepressant)	Antioxidant properties
Harvey et al. (77); Lotter et al. (78)	Raw mangosteen pericarp (50 mg/kg)	↓IL-6 and TNF-α ↓ cortico-striatal lipid peroxidation	*In vivo* inflammatory rat model of schizophrenia *cf*. haloperidol	Antioxidant and Anti-inflammatory properties
Wang et al. (66)	α-Mangostin, 8-Deoxygartanin, Gartanin, Garciniafuran, Garcinone C, Garcinone D, and γ-Mangostin	↓β-amyloid build up ↓ DPPH ↑ROS scavenging (↓ oxidative stress) ↑neuroprotective properties	*In vitro*	Antioxidant and Neuroprotective properties
Catorce et al. (79)	α-Mangostin	↓IL-6 and COX-2 ~IL-β and TNF-α,	*In vivo* 18 mice with LPS induced neuroinflammation.	Anti-inflammatory properties
Gutierrez-Orozco et al. (80)	α-mangostin	↓IL-8 and TNF-α ↑ TNF-α in monocyte-derived macrophages cells	*In vitro*. LPS-induced inflammation in human cells.	Anti-inflammatory properties
Tewtrakul et al. (81)	Mangosteen pericarp (ethanoic extract) α-mangostin γ-mangostin	↓NO, PGE_2_, TNF-α, IL-4 ↓iNOS (α- and γ-mangostin) ↓ COX-2 (α-mangostin only)	*In vitro*. LPS-induced inflammation in murine RAW264.7 macrophage cells.	Anti-inflammatory properties
Chen et al. (82)	α- and γ-mangostin (ethyl acetate extract)	↓NO, PGE_2_ ↓ iNOS ~ COX-2	*In vitro*. LPS-induced inflammation in murine RAW264.7 macrophage cells.	Anti-inflammatory properties
Cho et al. (68)	Mangostenone F	↓ NO, TNF-α, IL6 and IL-1β, ↓ iNOS ↓ NF-κB (via p65 and IκB-α) ↓ MAPK (vis AP-1)	*In vitro*. LPS-induced inflammation in murine RAW264.7 macrophage cells.	Anti-inflammatory properties
Bumrungpert et al. (83)	α- and γ-mangostin	↓IL-6, IL1β, interferon-γ and TNF-α ↓ MAPK, NF-κB	*In vitro*	Anti-inflammatory properties
Hu et al. (84)	α-mangostin (1, 10, and 100 nM).	↓ IBA-1 and iNOS production ↓ H_2_O_2_ (reduced ROS) ↑ Dopamine uptake	*In vitro*—wild-type Sprague-Dawley rat cells treated with α-synuclein induced inflammation.	Anti-inflammatory properties
Weecharangsan et al. (85)	Mangosteen pericarp extracted by: Water vs. 50% ethanol vs. 95% ethanol vs. ethyl acetate	↓ DPPH free radical scavenging. ↓NG108-15 (water and 50% ethanol superior) ↓ H_2_O_2_ Cell death	*In vitro* NG108-15 cells treated with H_2_O_2_	Neuroprotective and antioxidant properties
Janhom et al. (86)	α-mangostin	↓ apoptosis and ROS ↓Bax, Bax/Bcl-2, p53, and caspase 3 ↑ Bcl-2	*In vitro* human SH-SY5Y neuroblastoma cells in an MPP^+^ Parkinson's Disease like state	Neuroprotective and anti-apoptotic properties
Phyu and Tangpong (87)	Xanthones from aqueous extract of mangosteen pericarp	↓acetylcholinesterase ↓MDA	*N* = 42 lead-poisoned mice	Antioxidant and neuroprotective properties
Sattayasai et al. (88)	Mangosteen pericarp extract	↓ROS	*In vivo* and *In vitro* memory impaired mice	Antioxidant and neuroprotective properties
Wihastuti et al. (89)	Mangosteen pericarp extracted by ethanol solution	↓ VEGFR-1, NF-κB	*In vivo* 20 male rats-−5 groups: Normal diet High cholesterol diet High cholesterol and mangosteen pericarp 200 mg/kg High cholesterol and mangosteen pericarp 400 mg/kg High cholesterol and mangosteen pericarp 800 mg/kg	Neurogenesis, anti-oxidative and anti-inflammatory properties
Huang et al. (90)	Mangosteen pericarp extract	~ JNK, ERK ↓ROS, COX-2 and IL-6 ↑GSH, brain derived neurotropic factor, and serotonin	*In vivo* and *In vitro*: 3xTg-AD mouse model of Alzheimer's Disease. Hippocampal cells and serum.	Antioxidant and neuroprotective properties
Jariyapongskul et al. (91)	α-mangostin	↓ VEGF, TNF-α, MDA, and fasting glucose	*In vivo*: 56 type 2 diabetic rats, retinal blood.	Neurogenesis, anti-inflammatory, antioxidant, and anti-hyperglycemic properties
Aisha et al. (92)	combination of α- and γ-mangostin (81 and 16%, respectively)	↑caspases-3/7 ↑MAPK, ERK and p52	*In vitro*: human colon cancer cells	Activate mitochondrial pathway of apoptosis
Tang et al. (93)	Commercially available Mangosteen juice (Mangosteen Plus^TM^ with Essential Minerals®), main xanthone: β-mangostin	↓IL-1α ↓CRP ~ IL-β and IL-2	Randomized, double-blind, placebo-controlled trial (*n* = 60)	Anti-inflammatory properties
Xie et al. (94)	Mangosteen-based drink (Verve®)	↓Peroxyl radical scavenging capacity (antioxidant activity) ↓CRP ~ IL- 1α, IL- 1β, and IL- 2	Randomized, double-blind, placebo-controlled trial (*n* = 60)	Antioxidant and anti-inflammatory properties
Udani et al. (95)	Mangosteen juice from whole fruit, combined with other fruit juices (XanGo Juice^TM^)	↓CRP (18 oz/day group only), IL-12p70 ↓ Body mass index (for 6oz group only) ~ENA-78 and lipid peroxidation	Randomized, double blind placebo-controlled pilot of obese participants (*n* = 40)	Anti-inflammatory properties

*3-NP, 3-nitropropionic acid; Bcl-2, B-cell lymphoma 2; COX-2, cyclooxygenase-2; CRP, C-reactive protein; DPPH, 1,1-diphenyl-2-picrylhydrazyl; ENA, epithelial Cell-Derived Neutrophil-Activating Protein; ERK, extracellular Signal-regulated Kinase; GPx, glutathione peroxidase; GSH, glutathione peroxidase; H_2_O_2_, hydrogen peroxide; IBA-1, ionized calcium binding adaptor molecule 1; IL, interleukin; iNOS, inducible nitric oxide synthase; JNK, c-Jun N-terminal; LPS, lipopolysaccharide; MAPK, Mitogen-activated protein kinases; MDA, malondialdehyde; NF-kB, nuclear factor kappa B; NO, nitric oxide; PGE_2_, prostaglandin E_2_; ROS, reactive oxygen species; SOD, superoxide dismutase; TBARS, thiobarbituric acidic reactive substances; TNF-α, tumor necrosis factor; VEGF, vascular endothelial growth factor*.

**Figure 1:**
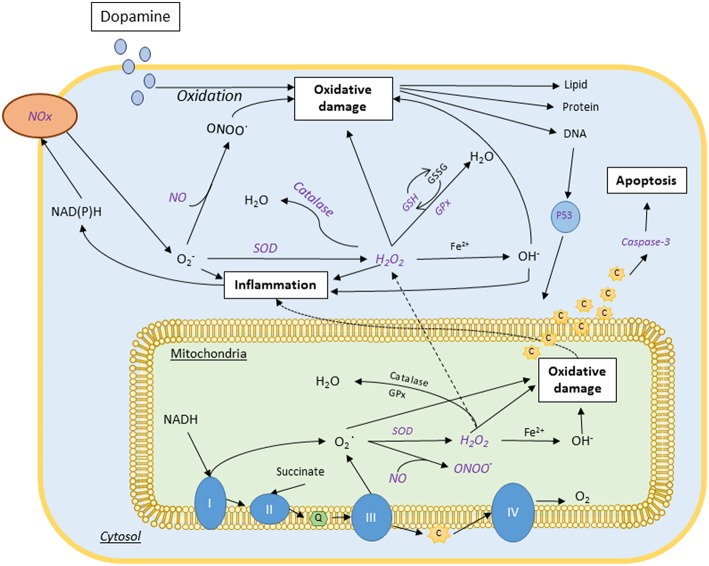
Proposed neurobiology of bipolar disorder and schizophrenia and associated mechanisms of mangosteen pericarp (purple italics). These major psychiatric disorders have been shown to have aberrations in oxidative biology, mitochondrial function and neurogenesis/apoptosis. The purple italicized text indicates the points at which mangosteen pericarp has mechanistic actions that may benefit these disorders. Complexes I, II, II, and IV; CoQ, Coenzyme Q; C, cytochrome C; Fe^2+^, ferrous ion; GPx, glutathione peroxidase; GSH, reduced glutathione; GSSG, oxidized glutathione; H_2_O, water; H_2_O_2_, hydrogen peroxide; NADH, nicotinamide adenine dinucleotide (phosphate); NO, nitric oxide; Nox, NADH, Reduced Nicotinamide adenine; O2, Superoxide anion; OH, Hydroxyl radical; ONOO, peroxynitrite; SOD, Superoxide dismutase.

To further illustrate the biomarkers and mechanistic pathways mangosteen pericarp may have an effect on, Figure 1 provides a summary of the molecular pathways implicated in oxidative stress, inflammation and mitochondrial function and which may be targeted by mangosteen pericarp.

### The Neuroreceptor Profile of Mangosteen Pericarp Extract

Studies have demonstrated that α- and γ-mangostin have anti-histaminergic properties and can selectively block serotonin type 2A (5-HT_2A_) receptors in rabbit aorta, a pathway that is a feature of some atypical antipsychotics (96, 97). Furthermore, α- and γ-mangostin have demonstrated some inhibitory effects on cyclic adenosine monophosphate (cAMP) phosphodiesterase (98, 99), a property shared with another putatively psychoactive plant, sceletium tortuosum (100). Indeed, cyclic adenosine monophosphate cAMP phosphodiesterase inhibitors, such as rolipram, have antidepressant and anti-inflammatory activity (101). Targeting these pathways is implicated in antidepressant and antipsychotic treatments (97).

### Antioxidant Properties of Mangosteen Pericarp

Marquez-Valadez et al. (72) explored α-mangostin as a treatment to reduce oxidative damage in homogenized rat brain tissue (cerebellum removed) and synaptosomal P2 fractions, in a model of neurotoxicity. Following administration of various neurotoxins, *viz*. ferrous sulfate, quinolinic acid, and 3-nitropropionic acid (3-NP), administration of α-mangostin (25–500 uM) resulted in a reduction in toxin-induced oxidative stress as measured by TBARs formation, with all doses being effective. α-Mangostin also reduced quinolinic acid and 3-nitropropionic acid induced mitochondrial dysfunction as assessed by 3-(2,5-dimethylthiazol-1-yl)2,5-diphenyltetrazolium (MTT) reduction. It was concluded that α-mangostin was effective as a broad-spectrum antioxidant.

Marquez-Valadez et al. (73) then examined α-mangostin modulation of the GSH system and antioxidant properties in rat brain tissue prepared as above (72). Rats (*n* = 4–5 per group) received varying doses of α-mangostin (10, 25, and 50 μM) either alone or with ferrous sulfate or with 3-NP. Synaptosomal fractions were analyzed for glutathione (GSH), glutathione peroxidase GPx, and glutathione S-transferase (GST) levels. All doses of α-mangostin reduced GSH levels compared to controls when tested alone (no ferrous sulfate or 3-NP). In the ferrous sulfate studies, α-mangostin at doses of 25 and 50 μM returned GSH levels to control levels and were significantly higher than that of the ferrous sulfate group. Similar results were found with respect to GSH levels following 3-NP challenge for all doses of α-mangostin. GPx activity was increased only in the α-mangostin 25 and 50 μM doses compared to controls, but this effect was lost when administered alongside ferrous sulfate_._ There were no differences in glutathione S-transferase activity across any of the groups. Due to the varying effects of α-mangostin on redox activity, the authors concluded that α-mangostin was selectively modulating the GSH system to preferentially raise protective GSH levels, thereby highlighting a putative mechanism for α-mangostin's antioxidant properties.

Moongkarndi et al. (65) compared 25 μg/ml doses of purified α-mangostin with mangosteen pericarp extracts using two different solvents—ethyl acetate and water to explore the bioactive components in SKBR3 cells, a breast cancer cell line. The ethyl acetate-soluble extract, noted to contain low polar constituents, appeared to inhibit cancer cell proliferation. The purified α-mangostin and the water extract of mangosteen pericarp that contains high polar constituents both demonstrated antioxidant activity. In particular, the water-soluble extract demonstrated the most pronounced free-radical scavenging activity against 1,1-diphenyl-2-picrylhydrazyl (DPPH). It was concluded that purified α-mangostin showed superior activity in reducing cytotoxicity, apoptosis, and antioxidative activity in cancer cells, compared to the water extract. Previous cancer studies have shown mangosteen pericarp to be pro-apoptotic in certain laboratory conditions [e.g., Lee et al. (74) and Yang et al. (67)]. Differences in tissue type (e.g., cancer cells), dosing and other parameters complicate the interpretation of these studies within the context of neurobiology. Similarly, biological agents often have both beneficial and detrimental effects, dependent on these factors. For example, in an environment of oxidative stress, *N*-acetylcycteine has beneficial effects but can be toxic (due to oversupply of cysteine) under conditions of normal redox homeostasis (102).

Shin-Yu et al. (75) fed mangosteen pericarp extract (85% α-mangostin; 25 mg/day) to rats in addition to a high-fat diet and compared changes in oxidative stress and mitochondrial activity among rats fed a high fat diet and a group on AIN-93M control diet. Results showed significantly reduced liver TBARS levels in mangosteen fed rats compared with the high-fat diet group (and were similar levels to controls). The authors posited that the reduction in oxidative stress and increased cellular protection (measured by TBARS) could be due to mangosteen pericarp-induced increases in cellular oxidative defense mechanisms. All antioxidant enzymes explored were significantly higher in the mangosteen pericarp extract group than that described in the high-fat diet group (i.e., GSH, GPx, glutathione reductase, SOD, and catalase; CAT). This study also suggested the potential utility of mangosteen pericarp in a population that has high co-morbid obesity and metabolic disorders (30).

### Anti-inflammatory Properties of Mangosteen Pericarp

Catorce et al. (79) explored the anti-inflammatory properties of α-mangostin in a murine model. They administered lipopolysaccharide (LPS) to mice (*n* = 18) to induce neuroinflammation. Results showed that oral gavage administration of α-mangostin significantly inhibited the LPS-induced increase in IL-6 in the brain. The levels of other inflammatory cytokines studied (IL-1β and TNF-α) were not affected by α-mangostin administration. This study further demonstrated α-mangostin-associated reduction in the levels of the inflammation-associated enzyme COX-2, in the brain.

The anti-inflammatory effects of α-mangostin have also been observed in human cells challenged with LPS (80), where α-mangostin was found to significantly reduce the release of pro-inflammatory cytokines IL-8 and TNF-α. Interestingly, these results were only true for THP-1 (monocyte-like leukemia), HepG2 (hepatocellular carcinoma), and Caco-2 HTB-37 (colorectal adenocarcinoma with enterocyte-like phenotype) cells, but not for other cell-lines such as monocyte-derived macrophages. These results suggest the effects of α-mangostin may differ depending on cell type. In contrast, α-mangostin stimulated the release of TNF-α in monocyte-derived macrophages cells.

In a study by Tewtrakul et al. (81), an ethanolic extraction of mangosteen pericarp and α- and γ-mangostin isolations were administered to murine RAW264.7 macrophage cells to explore the pathway of anti-inflammatory action of the compounds. LPS was first used to produce an increase in inflammatory molecules NO, prostaglandin E_2_ (PGE_2_), TNF-α, and IL-4, with mangosteen pericarp and its isolates administered in different concentrations (0, 0.3, 1, 3, 10, 30, and 100 μM). Release of NO was significantly inhibited by α-mangostin (3, 10, 30, and 100 μM), and by γ-mangostin and mangosteen pericarp (10, 30, and 100 μM). Release of PGE_2_ was significantly inhibited by all compounds at all doses. Pro-inflammatory cytokine TNF-α release was inhibited by mangosteen pericarp (10 and 30 μM) and by α- and γ-mangostin (30 and 100 μM). All extracts significantly inhibited release of IL-4 (10, 30, and 100 μM). However, the inhibition of TNF-α and IL-4 were only of moderate effect. Lastly, inducible iNOS and COX-2 expression were inhibited by α-mangostin, with γ-mangostin only inhibiting iNOS. Similar studies found an ethyl acetate extract of α- and γ-mangostin inhibited LPS induced NO and PGE_2_ production and iNOS (but not COX-2) expression in murine RAW264.7 cells (82). Therefore, the authors concluded that mangosteen pericarp and, in particular, α- and γ-mangostin have potential anti-inflammatory activity.

Cho et al. (68) also investigated the effects of the xanthone, mangostenone F, an isolated compound of the mangosteen pericarp, on LPS-induced inflammation in murine RAW264.7 macrophage cells. The RAW264.7 cells were pre-treated with mangostenone F (0, 10, 20, 30, 40, 60, 80, and 100 μM) for 24 h. Mangostenone F significantly inhibited the production of NO in a dose dependent manner by decreasing the expression of iNOS. To explore the effects of mangostenone F on the pro-inflammatory cytokines TNF-α, IL6, and IL-1β, the cells were pre-treated at doses of 20, 40, and 60 μM. There was a dose dependent reduction in all pro-inflammatory cytokines by mangostenone F. There was also a dose dependent reduction in NF-κB DNA binding activity, via p65 and IκB-α. Lastly, AP-1 reporter activity was inhibited by the mangostenone F, suggesting suppression of the MAPK signaling pathway. Therefore, it was suggested that the anti-inflammatory response was via suppression of MAPK and NF-κB activation. In agreement with afore-noted findings, an *in vitro* study in human cells examined whether α- and γ-mangostin could reduce obesity-associated inflammation (83). The study found that the reduction in inflammation was possibly due to the mangosteen pericarp extract preventing MAPK and NF-κB activation which in turn reduced levels of IL-6, IL-1β, interferon-γ and TNF-α (83).

A study investigating a cell culture model of Parkinson's disease included investigations of NO and iNOS response to α-mangostin (84). There was a significant dose-dependent reduction of iNOS by α-mangostin, showing the effects of α-mangostin to reduce immunologically-induced NO release. The authors also explored the NF-κB signaling pathway via IκB-α and p65 in the cytosol and found α-mangostin had a concentration dependent beneficial effect on these pathways. Therefore, this may be a pathway for α-mangostin reduction of pro-inflammatory cytokines and NO production. In addition, they noted that ROS was significantly reduced by α-mangostin in a dose dependent manner in microglial cells, demonstrated by reduction of H_2_O_2_. The authors posited that this may be due to α-mangostin targeting NADPH-oxidase (NOX). Reduced dopamine uptake induced by α-synuclein was also increased by α-mangostin, with α-mangostin significantly protecting dopamine neurons from apoptosis in a dose-dependent manner. It was concluded that α-mangostin has demonstrated capacity as a neuroprotective agent in neurodegenerative disorders via microglial activation pathways of neuroinflammation and serves as an anti-inflammatory and antioxidant agent.

Production of NO through iNOS and inflammatory process have been implicated in both psychosis and depression (23, 103). It is relevant to note that diverse antidepressants (104) and antipsychotics (105) target the NO system, while selectively targeting the NO system has been implicated in the antidepressant and antipsychotic actions of methylene blue (62, 63).

### Neuroprotective and Anti-apoptotic Properties of Mangosteen Pericarp

Effective neuroprotective compounds will impede or stop the progression of an illness (44). Neuroprotective compounds can modulate antioxidant systems (85) and inflammatory systems (44). Weecharangsan et al. (85) investigated the neuroprotective properties of four mangosteen pericarp extractions: distilled water, 50% ethanol, 95% ethanol or ethyl acetate. Each treatment group was assessed for antioxidant activity through DPPH free radical scavenging and for neuroprotective activity in NG108-15 cells treated with hydrogen peroxide (H_2_O_2_). Both the water and 50% ethanol extracts dose-dependently exhibited superior free radical-scavenging activities and inhibited H_2_O_2_-induced cell death, compared to the other extracts.

Xanthones extracted from mangosteen pericarp (aqueous extraction) were explored for neuroprotective properties in lead-poisoned mice (87). Lead results in cognitive impairments by inhibiting antioxidant function and increasing free radical production. This is achieved by lead competitively inhibiting calcium binding sites on acetylcholinesterase, leading to oxidative damage. Xanthone treatment (administered orally, in drinking water) had a significant dose-dependent effect on increasing acetylcholinesterase activity in the blood and brain of lead-treated mice. Oxidative stress in the mangosteen pericarp treatment groups was significantly reduced as shown by MDA reduction. Thus, the authors concluded that the xanthone component of mangosteen pericarp has neuroprotective properties while reducing cognitive impairment by inhibiting oxidative stress. In addition to these results, depressive-like behavior in lead-intoxicated mice as demonstrated using the forced swim test was significantly reversed by the xanthone extract group compared to control groups (87).

In an *in vivo* study by Wihastuti et al. (89), the effect of mangosteen pericarp on neurogenesis was explored. Varying doses (200, 400, and 800 mg/kg) of mangosteen pericarp extracted by an ethanol solution were trialed via gavage in rats fed a high-cholesterol diet (and compared to a normal diet, negative control group and high-cholesterol diet, positive control group). The VEGF receptor 1 and NF-κB were measured. VEGF receptor 1 is expressed in inflammatory cells including macrophages and monocytes. The protein NF-κB responds to free radicals and is involved in the production of cytokines and influences synaptic plasticity and memory. Mangosteen pericarp extract significantly inhibited the formation of VEGF receptor 1, and reduced NF-κB, iNOS, H_2_O_2_, and H1F1-α expression. The highest dose (800 mg/kg) was most effective in terms of anti-oxidative and anti-inflammatory activities.

Huang et al. (90) investigated the effects of a mangosteen pericarp extract rich in xanthones and polyphenols in a 3xTg-AD mouse model of Alzheimer's Disease to explore neuroprotective and anti-apoptotic properties. Mice received the mangosteen pericarp extract in addition to a regular diet and were compared to a control group fed only the regular diet. Analyses were conducted both *in vitro* in hippocampal cells and *in vivo* with respect to serum markers. In hippocampal cells, no differences in JNK or ERK pathways were noted in the hippocampal cells, although an increase in GSH levels was observed. However, the results in serum showed a reduction in ROS, cyclooxygenase-2 (COX-2), and IL-6, as well as an increase in GSH and serotonin. Lastly, mice treated with the mangosteen pericarp dietary supplement presented with reduced cognitive impairment and spatial memory retrieval deficit compared to untreated controls. It was concluded the mangosteen pericarp extract demonstrated antioxidative, anti-inflammatory and neuroprotective properties.

To test for anti-inflammatory, antioxidant, and anti-hyperglycemic properties of the mangosteen pericarp, Jariyapongskul et al. (91) investigated the effects of α-mangostin on inflammatory cytokines, oxidative stress markers, and neurotrophins in the retina of streptozotocin-induced diabetic mice. In an *in vivo* study, within 8 weeks of intraperitoneally injecting rats with streptozotocin, α-mangostin was administered via gavage to type-2 diabetic rats (*n* = 56). The treatment with α-mangostin reduced ocular degeneration, a manifestation that can occur in early stages of type 2 diabetes. The authors found that α-mangostin treatment reduced levels of VEGF, TNF-α, and MDA. Whilst α-mangostin significantly reduced fasting glucose levels of the diabetic rats, there was no difference between non-diabetic rats and control rats, suggesting a role in glucose regulation. Whilst this study illustrates some promise as a treatment option in type 2 diabetes, further research is needed to determine if it may also be a preventative strategy for the disorder and for the development of vascular abnormalities. Due to the relationship between general medical conditions, including metabolic disorders such as diabetes and bipolar disorder and schizophrenia, this study highlights the potential symbiosis of treating the disorders together (41).

### Mitochondrial Enhancing Properties of Mangosteen Pericarp

Many of the biological targets for mangosteen pericarp and its isolates involve the mitochondria. For example, when mitochondrial dysfunction was induced *in vitro* via 3-NP, increased oxidative stress was mitigated by α-mangostin (72, 73, 75). It was further suggested that α-mangostin may modulate apoptosis associated with mitochondrial pathways (86). This shows how complex and inter-related the mitochondrial, oxidative stress, and inflammation pathways are, and suggests the potential of treatments that can target these pathways.

To better inform the therapeutic potential of mangosteen pericarp in psychiatry, we investigated other fields where mangosteen pericarp has been shown to have relevant actions. Cancer cells studies can demonstrate the relationship between apoptosis and the mitochondria, relevant to psychiatry. Aisha et al. (92) explored the anti-colon cancer effects of a combination of α- and γ-mangostin (81 and 16%, respectively) through *in vitro* and *in vivo* experiments. For the *in vitro* study, human colon cancer cells were treated with the xanthone extract of mangosteen pericarp and α-mangostin and compared to the chemotherapy medication, cisplatin as a positive control. The treatment with xanthones killed the cancer cells and did so at a lower concentration than cisplatin. Their findings suggested an action mediated by enhancing executioner caspases-3/7 and by activating the initiator caspase-9 leading to apoptosis of cancer cells. The authors postulated that mitochondria-mediated cytotoxicity was involved. Apoptosis was further analyzed through the upregulation of the MAPK/ERK and p53 pathways, showing potent, selective, and dose dependent cytotoxicity due to the enhancement and activation of mitochondrial pathways of apoptosis.

This review outlines pathways implicated in oxidative stress, inflammation and mitochondrial function. Importantly, these pathways are also directly or indirectly linked to monoamine release and/or function. As previously mentioned, α-mangostin has a tricyclic structure (64) which is relevant to existing medications (e.g., tricyclic antidepressants) which block serotonin re-uptake. However, we believe that because mangosteen has other important biological properties in addition to potentially modulating monoamines, mangosteen pericarp may be a novel and indeed highly efficacious adjunctive therapy.

## Therapeutic Potential of Mangosteen Pericarp

### Mangosteen Pericarp as an Antidepressant

Recent preclinical evidence has demonstrated the antidepressant and memory enhancing actions of mangosteen pericarp, together with a suppression of hippocampal lipid peroxidation, in a rodent model of depression (76). In this study, an extract of mangosteen pericarp at an acute dose of 50 mg/kg administered by oral gavage was found to be an effective antidepressant in Flinder's Sensitive Line rats (a model of depression). The raw pericarp extract contained predominantly α- and γ-mangostin. A 14-day treatment regimen of mangosteen pericarp extract (50 mg/kg per day) displayed sustained antidepressant and pro-cognitive effects in the forced swim test and novel object recognition test, respectively, while demonstrating parity with the reference tricyclic antidepressant, imipramine (76). Behavioral and regional brain monoamine assessments suggested a more prominent serotonergic action for mangosteen pericarp extract as opposed to the noradrenergic action of imipramine, with both imipramine and mangosteen pericarp extract reversing hippocampal lipid peroxidation in rats. Indeed, the hippocampus is highly vulnerable to oxidative stress while being a key factor in memory. Moreover, both memory and hippocampal structure and function are compromised in patients with depression (106). This work confirms the antidepressant activity of raw mangosteen pericarp, while linking this therapeutic action to correction of disordered brain monoamines as well as the restoration of cellular damage brought on by oxidative stress (76). Interestingly, disordered redox status is known to mediate changes in brain monoamines (107) that in turn can drive changes in behavior (108).

### Mangosteen Pericarp as an Antipsychotic

Concerning schizophrenia, preclinical findings in a maternal immune-activation (MIA) rat model of schizophrenia found that chronic oral dosing of haloperidol (2 mg/kg for 14 days) and raw mangosteen pericarp (50 mg/kg for 14 days) were equally effective in reversing MIA-induced deficits in sensorimotor gating and depressive-like behavior, with haloperidol plus mangosteen showing a more pronounced response (77, 78). MIA-induced elevations in IL-6 and TNF-α levels and cortico-striatal lipid peroxidation were reversed by haloperidol, mangosteen, and haloperidol plus mangosteen. The authors suggested that, at least in this model, depressive manifestations are more responsive to mangosteen than sensorimotor gating deficits, implicating promise in the management of mood-related deficits in schizophrenia (77, 78).

### Mangosteen Pericarp as a Treatment for Neurodegenerative Disorders

The neuroprotective effects of α-mangostin were investigated in a cellular model of Parkinson's disease (86) by exposing human SH-SY5Y neuroblastoma cells to 1-methyl-4-phenylpyridinium (MPP^+^). α-mangostin was then administered for 24 h (doses 2.5, 5, 10, 20, and 40 μM). Doses of 20 and 40 μM α-mangostin induced significant loss of cell viability and were excluded from further experiments. All other doses of α-mangostin significantly decreased ROS induced apoptosis in an MPP^+^ model designed to trigger apoptosis. α-mangostin also significantly reduced MPP^+^ induced Bax, Bax/Bcl-2, p53, and caspase-3 expression. Therefore, the study suggests the ability of α-mangostin to reduce apoptosis, potentially via mitochondrial pathways and reduction of oxidative stress. Of interest, Parkinson's disease has been viewed as a biological parallel to bipolar disorder because of the dopaminergic pathology and the cyclical nature of depression which occurs in the on-off phenomenon in Parkinson's disease (11). In fact, novel methylene blue analogs which inhibit nitric oxide synthase (NOS) and inhibit monoamine oxidase have been synthesized to address such a comorbid condition by virtue of their neuroprotective actions and restoration of mitochondrial function (62).

In the study utilizing a Parkinson's disease model, the effects of α-mangostin on neuroinflammation via the microglial activation pathway was investigated (84). In this study, wild-type Sprague-Dawley rat cells were treated with α-synuclein to induce inflammation and then treated for 24 h with α-mangostin at 1, 10, and 100 nM doses. Results showed a significant dose dependent reduction in pro-inflammatory cytokines IL-6, IL-1β, and TNF-α in the α-mangostin treated group. The 100 nM dose of α-mangostin reduced microglial activation by inhibiting production of a marker of ionized calcium binding adaptor molecule 1(IBA-1), a microglial specific protein.

Mangosteen pericarp has been investigated as a treatment for Alzheimer's disease (66). In one *in vitro* study, the seven most common xanthones were isolated (α-mangostin, 8-deoxygartanin, gartanin, garciniafuran, garcinone C, garcinone D, and γ-mangostin) and assessed for their ability to inhibit β-amyloid-related cell damage, as well as their metal chelating, antioxidant and neuroprotective properties in an Alzheimer's Disease model (66). Results demonstrated that mangosteen pericarp reduced β-amyloid build up and reduced glutamate-induced cell damage by scavenging ROS (assessed by DPPH). Of the isolated xanthones, α-mangostin, gartanin, garcinone C, and γ-mangostin showed the greatest antioxidant properties.

Similar protective effects of mangosteen pericarp extract were explored *in vitro* and *in vivo* in mice in a study by Sattayasai et al. (88). Mice were administered scopolamine to induce memory impairments in an attempt to model the cognitive symptoms of Alzheimer's disease through central cholinergic muscarinic receptor antagonism. Afflicted mice were administered either mangosteen pericarp extract at 100 mg/kg via oral gavage or water as a control. Results obtained using the Morris water maze test for spatial memory and passive avoidance (fear) tests showed mangosteen pericarp extract protected mice from the memory degrading effects of scopolamine, leading to improved memory retention. In the *in vitro* arm of the study, mangosteen pericarp was protective against H_2_O_2_ and polychlorinated biphenyl induced oxidative stress in SK-N-SH (human blastoma cells) cells pre-incubated with mangosteen pericarp extract as shown by reduced ROS. This study demonstrated not only the anti-oxidative and neuroprotective properties of mangosteen pericarp extract, but also its memory protecting capacity, and thus is congruent with the *in vivo* depression model data described in Flinder's Sensitive Line rats by Oberholzer et al. (76).

## Clinical Trials of Mangosteen Pericarp

Mangosteen pericarp, as well as isolated compounds such as α-mangostin, have been demonstrated in both animal and *in vitro* studies to favorably modulate pathways relevant to mitochondrial function, inflammation, and oxidative stress. However, clinical trials are required to confirm whether these properties are clinically relevant. The following sections will provide an overview of the existing clinical trial data and its relevance to psychiatry, in particular bipolar disorder and schizophrenia. The behavioral effects of mangosteen pericarp are summarized in Table 2.

**Table 2:** Summary of behavioral evidence for *Garcinia mangostana Linn*. (mangosteen).

**Paper**	**Mangosteen compound**	**Outcome**	**Method**
Oberholzer et al. (76)	Mangosteen pericarp extract	↓ Depressive-like behaviors, ↑ Recognition memory	*In vivo*: Flinders sensitive line rats, compared with imipramine (tricyclic antidepressant)
Harvey et al. (77); Lotter et al. (78)	Raw mangosteen pericarp (50 mg/kg)	↓ Depressive-like behaviors	*In vivo* inflammatory rat model of schizophrenia cf. haloperidol
Phyu et al. (87)	Xanthones from aqueous extract of mangosteen pericarp	↓ Depressive-like behaviors	*In vivo* lead-poisoned mice (*n* = 42)
Sattayasai et al. (88)	Mangosteen pericarp extract	↑ Memory	*In vivo* and *In vitro* memory impaired mice
Huang et al. (90)	Mangosteen pericarp extract	↓ Cognitive impairment and spatial memory recall	*In vivo* and *In vitro*: 3xTg-AD mouse model of Alzheimer's Disease. Hippocampal cells and serum.
Chang et al. (109)	Mangosteen-based juice blend (containing 305 mg of α-mangostin and 278 mg of hydroxycitric acid)	~ Physical fatigue, heart rate, ↓ Mental fatigue	Randomized, double-blind, placebo-controlled trial healthy adults (*n* = 12)
Watanabe et al. (110)	Mangosteen pericarp (40% α- and γ-mangostin)	↓ Insulin levels and insulin resistance ~ Glucose levels, weight loss, waist circumference, body composition, LDL, HDL, triglycerides	Randomized controlled pilot study (*n* = 20)
Kudiganti et al. (111)	Meratrim (Sphaeranthus indicus flower and mangosteen pericarp at 3:1 ratio)	↓ Total mood disturbance	Randomized, double-blind, placebo-controlled trial in healthy overweight subjects (*n* = 60)
Laupu (112)	1,000 mg/day Mangosteen pericarp	↓ Depressive-like behaviors, positive and negative symptoms of schizophrenia, ↑ Life satisfaction and general functioning	Randomized, double-blind, placebo-controlled trial in schizophrenia/schizoaffective population (*n* = 80)

### Use of Mangosteen Pericarp in General Health

Mangosteen pericarp has been investigated in general medicine. Given these data are predominantly in healthy individuals, caution needs to be taken regarding the specific applicability to psychiatric disorders. However, to provide a comprehensive overview of the potential mechanisms by which mangosteen pericarp may be beneficial for psychiatric disorders these data have been included in this review.

A randomized, placebo-controlled, double blind trial studied the effects of 30 days of treatment with a commercially available mangosteen juice (Mangosteen Plus^TM^ with Essential Minerals®; mixed with vitamins A, B-6, C, D, E, selenium, folate, and thiamine; 59 ml) on immunity in 60 healthy human participants aged 40–60 years (93). Fructose liquid (59 ml) was used as a placebo control. The most prominent bioactive substances in the juice were β-mangostin and catechins. The mangosteen juice group had significantly higher levels of inflammatory cytokines IL-1α and IL-1b compared to placebo. To note, anti-inflammatory medications are adept at reducing heightened inflammation. If there is no inflammation in the system then adding anti-inflammatories may be detrimental, or even toxic (e.g., as with *N*-acetylcycteine (102). The mangosteen juice group also reported a reduction in inflammatory biomarker C-reactive protein (CRP) compared to baseline. There were no significant group differences with respect to IL-1β and IL-2. Interestingly, all participants in the mangosteen juice group self-reported an increase in subjective health status compared to the placebo group.

A randomized, double-blind, placebo controlled clinical trial explored antioxidant and anti-inflammatory biomarkers in healthy adults who were administered a mangosteen-based drink (94). Whilst mainly containing mangosteen, the drink also included vitamins, green tea, aloe vera, and a caffeinated energy blend. A total of 60 adult participants (30 men, 30 women) were administered 245 ml of either the mangosteen-based drink or placebo (fructose liquid) daily for 30 days together with pre- and post-administration blood analyses. After 30 days, results showed significantly more antioxidant activity, as measured by an increase in the peroxyl radical scavenging capacity, in the mangosteen-based drink group compared to the placebo arm. CRP levels significantly decreased in the mangosteen-based drink group and were not changed in the placebo group. There was no significant change in immunity markers IgA, IgG, IgM, C3, C4; and no significant change in inflammatory markers IL- 1α, IL- 1β, and IL- 2 across groups and time points.

Udani et al. (95) conducted a randomized, double-blind, controlled pilot study of commercially available mangosteen juice (XanGo Juice^TM^), a whole fruit juice blended with other fruit juices, in obese participants. A combination of fruit juices and sucrose was used as a control. A total of 40 participants who agreed to not change any current diet or exercise regimes and ceased any anti-inflammatory agents completed the study, all of whom were obese and had an elevated CRP score of ≥3. This was a 4-arm study: control, 3, 6, or 9 oz XanGo^TM^ juice whereby participants drank the intervention or control juice twice a day for 8 weeks. The combination juice used as a control was added to each of the lower mangosteen doses so each total individual serving volume was 9 oz liquid. As a result, participant received total daily doses of XanGO^TM^ juice at 6 oz, 12 oz or 18 oz, or control juice. Results showed a non-significant increase in CRP for the placebo group as well as a non-significant decrease in CRP for all doses of the mangosteen juice. There was a significant difference between changes in CRP across the 8 weeks in the control and the 18 oz/day group. Changes in body mass index and body fat were only significantly reduced in the 6 oz/day group compared to placebo. There were no significant differences between groups for lipid peroxidation and Epithelial Cell-Derived Neutrophil-Activating Protein (ENA)-78. There was a significant reduction of IL-12p70 levels across time in all active groups.

Due to the association between ROS and fatigue during exercise, Chang et al. (109) trialed mangosteen in 12 healthy adults. Participants were randomized to receive an acute dose of either a mangosteen juice blend or a diluted drink that replaced 50% mangosteen juice with water. There was no significant difference in time to exhaustion or other measures of physical performance (e.g., heart rate).

Mangosteen pericarp has been trialed in a combination treatment for weight loss in two human trials (111, 113) and mangosteen pericarp alone in one insulin resistance study (110) and all showed some promising results. In a population of obese female adults with insulin resistance (but not diabetes) mangosteen pericarp was trialed as a treatment for insulin resistance, reducing inflammatory markers and participant weight in a 26-week randomized controlled pilot study (110). All participants in the study (*n* = 20) received a lifestyle intervention delivered by a dietician focusing on physical activity and caloric restriction. Participants were randomized to receive either 400 mg/day mangosteen pericarp (40% α- and γ-mangostin) in addition to the intervention or no additional study medication. Participants in the mangosteen pericarp group showed significantly reduced insulin levels and demonstrated reduced insulin resistance. However, there were no significant differences in body fat percentage, waist circumference, or weight loss between participants in the mangosteen pericarp arm and control arm. There were no significant differences in glucose markers or in cholesterol markers and triglycerides levels when comparing the mangosteen pericarp and control groups. This study would have benefited from a placebo control to include blinding and reduce placebo response from the mangosteen administration. Given the lack of placebo, small sample size and female only sample, results from this study are cautiously interpreted as showing some efficacy in reducing insulin and insulin resistance and appears to be well tolerated.

These trials provide preliminary clinical evidence to suggest that mangosteen juice and mangosteen pericarp extract can alter inflammatory markers *in vivo*. However, due to most studies providing mangosteen in combination with other bioactive compounds, further trials are required to determine the effect of mangosteen pericarp or mangosteen juice as a standalone intervention for inflammation. Furthermore, the small sample sizes and predominately healthy populations included in these studies suggest that they may be underpowered.

It has been highlighted that the anti-inflammatory and antioxidant properties of varying extracts of mangosteen pericarp can also help to reduce co-morbid metabolic disorders common in those with bipolar disorder (114). Shandiz et al. (114) discussed this in their review on the metabolic effects of mangosteen pericarp extract *in vitro* and *in vivo*. Their review concluded that the reduction in metabolic disorders may occur by inhibiting inflammatory cytokines, reducing body weight and fat storage, and altering glucose metabolism.

### Use of Mangosteen Pericarp in Mental Health

In the study by Chang et al. (109) where the effect of mangosteen juice was explored in a small placebo-controlled trial for exercise fatigue in healthy adults, self-reported mood, and fatigue were assessed as a secondary outcome using the Profile of Mood States. There were no significant differences between depression scores of the mangosteen juice vs. placebo groups at any time point. Whilst both groups had an increase in fatigue following the exercise, those who received mangosteen had significantly less mental fatigue (measured on the Profile of Mood States scale) compared to the control intervention. Both groups also reported improvements in vigor and fatigue compared to baseline (109).

Mental health was assessed as a secondary outcome in a randomized controlled trial of a combination herbal treatment containing mangosteen pericarp (Meratrim®) in a healthy overweight human sample (111). The primary outcomes of the study were reduction in weight, body mass index, waist, and hip size which were all significantly improved in the Meratrim ® group compared to placebo. Participants (*n* = 60) were randomized to receive 400 mg, twice a day Meratrim (combination of Sphaeranthus indicus flower and mangosteen pericarp extract in a 3:1 ratio) or placebo. Participants receiving Meratrim reported reduced mood disturbances as measured by the Short form of the Profile of Mood States when compared to placebo.

In a double-blind placebo-controlled randomized trial, adjunctive mangosteen pericarp (1,000 mg) was investigated in participants with schizophrenia receiving second generation antipsychotic treatment (*n* = 80) (112). The mangosteen pericarp group performed significantly better than the placebo group across all outcomes including the primary outcome, the Positive and Negative Syndrome Scale and secondary outcomes including Montgomery Åsberg Depression Rating Scale, positive, negative, and general subscales of the Positive and Negative Syndrome Scale, Clinical Global Impression Severity and Improvement, Self-rated Life Satisfaction Scale, and Global Assessment of Functioning. Therefore, the study concluded there was a significant reduction in symptoms of depression and symptomatology of schizophrenia and schizoaffective disorder. The study was limited due to its small sample size, and while symptoms of depression were a secondary outcome, participants on average had mild depression at baseline as measured by the Montgomery Åsberg Depression Rating Scale. To date, this is the only study which directly assesses the potential of mangosteen pericarp at treating a serious mental illness. Due to the small sample size combined with promising results, this study provides significant impetus for further research of mangosteen pericarp for the treatment of bipolar disorder, schizophrenia, and other psychiatric disorders.

### Safety Profile of Mangosteen Pericarp

Whilst current clinical evidence is limited, several studies demonstrate that mangosteen pericarp appears to have a good safety profile and is well-tolerated. In animal models, mangosteen pericarp has been shown to reduce blood glucose levels, suggesting it could be used as a treatment for diabetes mellitus and that consumption of mangosteen pericarp may need to be supervised in patients undergoing insulin therapy (91, 115). In the insulin resistance study (110), gastrointestinal upset were the only reported adverse events and this occurred across both groups.

Suthammarak et al. (116) investigated the safety and antioxidant effects of mangosteen pericarp. Participants were orally administered polar (water-soluble) fractions of mangosteen pericarp in capsule form for 24 weeks. For the first three months, participants weighing under 55 kg received a 220 mg dose and those over 55 kg received 280 mg. After 3 months, all participants had their doses doubled. Participants were monitored at weeks 0, 1, 4, 12, 16, and 24. The study was limited by the lack of a placebo control group and a small sample size (*n* = 11) making it difficult to relate the emergence of adverse events to an association with the mangosteen pericarp. In addition, a small dose of mangosteen pericarp was used. Nevertheless, no major adverse events or medical issues were reported.

In a pilot study of 1,000 mg mangosteen pericarp for the adjunctive treatment of schizophrenia, there was no significant difference of reported adverse events between the placebo and the active groups (112). Only 2 adverse events were reported in this study (viz. headache and thoughts of self-harm). However, given the nature of the population, it is probable that adverse events could have been under-reported.

No adverse events were reported in a placebo-controlled randomized control trial of mangosteen juice in adults aged 40–60 years (93). Nor were there any adverse events in a similar study with a mangosteen-based drink (Verve®) (94). The mangosteen-based drink study also showed no significant difference compared to the placebo group for weight, body mass index, heart rate or blood pressure (94). Another mangosteen juice study again had no side effects reported and no clinically significant changes in electrocardiograms (95). In a study of 400 mg/day capsules of Meratrim for weight loss (*n* = 60), there was no significant difference in adverse events or liver, heart, kidney, or metabolic function, compared to the placebo group (113). This safety profile held true for another study of Meratrim at 800 mg/day (111). Interestingly, mangosteen pericarp has also been associated with increased renoprotection due to reduction in inflammation and oxidative and/or nitrosative stress (117). Hence, the current evidence suggests that mangosteen pericarp is well-tolerated with no known side effects. However, given the limited evidence base, particularly within clinical populations and those with polypharmacy, future trials are required to evaluate the long-term safety of this intervention across a range of doses and treatment durations. This is especially true when considering that the clinical application for mangosteen will be adjunctive to conventional treatments. With the recent study in animals suggesting increased serotonergic activity in mangosteen pericarp-treated depressed rats (76), and given the unknown interactions of mangosteen pericarp with conventional serotonergic agents, the possibility of drug-drug interactions should be considered.

### Use of Mangosteen Pericarp as an Adjunctive Treatment for Serious Mental Disorders

In summary, this review has summarized a number of properties of the mangosteen pericarp that could target known aberrations in bipolar disorder and schizophrenia. In terms of biological processes, bipolar disorder, and schizophrenia share heightened oxidative stress including an increase in ROS, RNS, TBARS, and MDA which may be modulated by the glutamatergic system. Mangosteen pericarp in animal models reduces ROS, TBARS, MDA, and has demonstrated effects on the glutamatergic system. Inflammation is present in bipolar disorder and schizophrenia indexed by inflammatory cytokines IL-6, IL-1Ra, IL-1β TNF- α, and also by NO production via iNOS, superoxide dismutase catalase, and glutathione peroxidase. Our review collates results showing mangosteen pericarp can also have an effect on IL-6, IL-1β TNF- α, NO, catalase, and glutathione peroxidase, in addition to IL-2, IL-8, COX-2, and NF-κB. Alterations in apoptosis and neurogenesis are demonstrated by changes in Ki67+ cells as well as relevant markers including VEGF, bcl-2, MAPK, and JNK. Mangosteen pericarp has demonstrated effects on VEGF, bcl-2, and MAPK. However, there were no significant demonstrated effects of mangosteen pericarp on JNK. Lastly, the mitochondrial disturbances observed in bipolar disorder and schizophrenia may be targeted by mangosteen via the mitochondrial pathway to apoptosis. In addition to the biological pathways, mangosteen pericarp has demonstrated potential in reducing depression which is a key phase in bipolar disorder and in negative symptoms of schizophrenia. However, future research is required to observe the efficacy of mangosteen pericarp across the scope of the disorders and in human participants.

## Future Directions

Mangosteen pericarp is a potential adjunctive treatment option in bipolar disorder and schizophrenia. Given only one randomized controlled trial has been completed in the field (112), future work could target the limitations of research such as the small sample sizes, lack of comparable outcomes, and non-standardization in the extraction process. Currently, a range of mangosteen pericarp extracts have been utilized which are either whole compound or isolated components (such as α- and γ-mangostin). Further research must be undertaken to discern the optimal dosing and extraction of the bioactive components, if separate, or if the bioactivity comes from a combination of the components working together in the compound. Mangosteen pericarp has been posited for use in neurodegenerative disorders such as Alzheimer's (66, 88, 90) and Parkinson's disease (84, 86). In animal models, mangosteen pericarp has been trialed for cognitive decline (87) and memory impairments (88). More recently, mangosteen pericarp displayed marked antidepressant and pro-cognitive effects in the Flinders Sensitive Line rat, a genetic animal model of depression (76). In addition, a reduction of hippocampal lipid peroxidation and correction of disordered regional brain monoamines and sensorimotor gating and depressive-like symptoms were reduced in an inflammatory rat model of schizophrenia (77, 78). Future directions could directly trial mangosteen pericarp as an adjunctive antidepressant in human trials for major depressive disorder and bipolar disorder. There are no animal studies utilizing the effects of mangosteen pericarp in a bipolar disorder model, however, due to the psychosis overlap with schizophrenia models, and the depressive phase of the illness, symbiotic benefits may be inferred. There is a need to demonstrate these findings from animal models in human studies. There are currently two studies directly trialing the effect of mangosteen pericarp on schizophrenia (118) (Trial registry ID: ACTRN12616000859482) and bipolar depression (119) (Trial registry ID: ACTRN12616000028404), both in adult populations.

## Conclusion

The evidence of the bioactivity and neurobiology of mangosteen pericarp is rapidly emerging. Mangosteen pericarp has produced promising results in animals, and has been demonstrated to have antioxidant, anti-inflammatory, anti-apoptotic, neuroprotective, and mitochondrial enhancing properties. Taken together, the theoretical biological rationale of psychiatric disorders, bioactivity of mangosteen pericarp extract and the available preclinical data, support the therapeutic potential as an adjunctive psychiatric treatment. As the clinical evidence base for mangosteen pericarp as an adjunctive psychiatric treatment is scarce, future research requires human clinical trials to explore the risks and benefits of treatment and assess the potential for translation into clinical care.

## Author Contributions

Initial planning of the paper was conducted by MA, OD, and MB. MA conducted the literature search and wrote the first draft. MA, CB, and AW created the descriptive figure. OD, AW, CB, CN, MH, BH, MM, JM, WM, AT, SD, JGS, J-PK, KW, JS, and MB contributed to and edited drafts of the paper.

### Conflict of Interest Statement

MB is a co-inventor on a patent application regarding the use of mangosteen and related compounds for psychiatric indications, assigned to Deakin University. MA has received grant/research support from Deakin University, Australasian Society for Bipolar Depressive Disorders, Lundbeck, Australian Rotary Health, Ian Parker Bipolar Research Fund, and Cooperative Research Center for Mental Health. MB has received grant support from NIH, Simons Autism Foundation, Cancer Council of Victoria, CRC for Mental Health, Stanley Medical Research Foundation, MBF, NHMRC, Beyond Blue, Geelong Medical Research Foundation, Bristol Myers Squibb, Eli Lilly, GlaxoSmithKline, Organon, Novartis, Mayne Pharma, and Servier. OD is a R.D. Wright Biomedical Research Fellow and has received grant support from the Brain and Behavior Foundation, Simons Autism Foundation, Stanley Medical Research Institute, Deakin University, Lilly, NHMRC and Australasian Society for Bipolar and Depressive Disorders (ASBDD)/Servier. CN had served as a consultant for Grunbiotics, Lundbeck, Servier, Janssen-Cilag, Wyeth and Eli Lilly, received research grant support from Wyeth and Lundbeck, and speaker honoraria from Servier, Lundbeck, Bristol-Myers Squibb, Organon, Eli Lilly, GlaxoSmithKline, Janssen- Cilag, Astra-Zenaca, Wyeth, and Pfizer. MH has received grant support from ISSCR, Servier, US DOD and Bionomics, has been a speaker for Janssen-Cilag, Lundbeck, and Servier, and has been a consultant for AstraZeneca, Eli Lilly, Janssen-Cilag, Lundbeck, and, Servier. SD has received grant support from the Stanley Medical Research Institute, NHMRC, Beyond Blue, ARHRF, Simons Foundation, Geelong Medical Research Foundation, Harry Windsor Foundation, Fondation FondaMental, Eli Lilly, Glaxo SmithKline, Organon, Mayne Pharma and Servier, speaker's fees from Eli Lilly, advisory board fees from Eli Lilly and Novartis and conference travel support from Servier. BH has participated in advisory boards and received honoraria from Servier,^®^ and has received research funding from Deakin University, Servier^®^ and Lundbeck,^®^and has received funding from Deakin University to specifically undertake mangosteen-related research in animal models. AT has received grants/research support from NHMRC, AMP Foundation, Schizophrenia Fellowship of NSW, the National Stroke Foundation, and the Hunter Medical Research Institute. JS has received either presentation honoraria, travel support, clinical trial grants, book royalties, or independent consultancy payments from: Integria Healthcare & MediHerb, Pfizer, Scius Health, Key Pharmaceuticals, Taki Mai, FIT-BioCeuticals, Blackmores, Soho-Flordis, Healthworld, HealthEd, HealthMasters, Kantar Consulting, Research Reviews, Elsevier, Chaminade University, International Society for Affective Disorders, Complementary Medicines Australia, SPRIM, Terry White Chemists, ANS, Society for Medicinal Plant and Natural Product Research, Sanofi-Aventis, Omega-3 Centre, the National Health and Medical Research Council, CR Roper Fellowship. J-PK has received research support, travel and educational support, consultancy payments, and/or presentation honoraria from Alkermes, AstraZeneca; Bionomics, Bristol-Myers Squibb; Eli Lilly; GlaxoSmithKline; Janssen; Lundbeck; Pfizer; Sanofi-Aventis; Servier; and Wyeth. MB has received Grant/Research Support from the NIH, Cooperative Research Centre, Simons Autism Foundation, Cancer Council of Victoria, Stanley Medical Research Foundation, MBF, NHMRC, Beyond Blue, Rotary Health, Meat and Livestock Board, Astra Zeneca, Woolworths, Avant and the Harry Windsor Foundation, book royalties from Oxford University Press, Cambridge University Press, Springer Nature and Allen and Unwin, has been a speaker for Astra Zeneca, Lundbeck, Merck and Servier and served as a consultant to Allergan, Astra Zeneca, Bioadvantex, Bionomics, Collaborative Medicinal Development, Grunbiotics, Janssen Cilag, LivaNova, Lundbeck, Merck, Mylan, Otsuka, and Servier. MB is a co-inventor on two provisional patents regarding the use of NAC and related compounds for psychiatric indications, assigned to the Mental Health Research Institute. The remaining authors declare that the research was conducted in the absence of any commercial or financial relationships that could be construed as a potential conflict of interest.

## Abbreviations

3-NP: 3-nitropropionic acid
Bcl-2: B-cell lymphoma 2
COX-2: Cyclooxygenase-2
CRP: C-reactive protein
DPPH: 11-diphenyl-2-picrylhydrazyl
ENA: Epithelial Cell-Derived Neutrophil-Activating Protein
ERK: Extracellular Signal-regulated Kinase
GPx: Glutathione peroxidase
GSH: Glutathione peroxidase
H_2_O_2_: Hydrogen peroxide
IBA-1: Ionized calcium binding adaptor molecule 1
IL: Interleukin
iNOS: Inducible nitric oxide synthase
JNK: c-Jun N-terminal
LPS: Lipopolysaccharide
MAPK: Mitogen-activated protein kinases
MDA: Malondialdehyde
MIA: Maternal immune-activation
MTT: 3-(2, 5-dimethylthiazol-1-yl)2,5-
NF-kB: Nuclear factor kappa B
NO: Nitric oxide
NOS: Nitric oxide synthase
NOX: NADPH-oxidase
PGE_2_: Prostaglandin E_2_
ROS: Reactive oxygen species
SOD: Superoxide dismutase
TBARS: Thiobarbituric acidic reactive substances
TNF-α: Tumor necrosis factor
VEGF: Vascular endothelial growth factor.
